# Condylar Sag After Orthognathic Surgery: Biomechanical Mechanisms, Surgical Factors, and Clinical Prevention

**DOI:** 10.7759/cureus.103519

**Published:** 2026-02-13

**Authors:** Bhavani M, G R Karthikeyan

**Affiliations:** 1 Department of Oral and Maxillofacial Surgery, Karpaga Vinayaga Institute of Dental Sciences, Kancheepuram, IND

**Keywords:** condylar sag, orthognathic surgery, proximal segment biomechanics, surgical fixation, temporomandibular joint adaptation

## Abstract

Condylar sag is a clinically important consideration following orthognathic surgery, with potential consequences for occlusal stability, skeletal relapse, and long-term temporomandibular joint (TMJ) health. Despite advances in digital surgical planning, fixation techniques, and imaging modalities, postoperative alterations in condylar position remain inconsistently defined, variably interpreted, and often managed without a unified conceptual framework. This narrative review synthesises contemporary literature (2000-2025) to address this gap by examining condylar sag from a biomechanical and functional perspective. The review focuses on the interaction between proximal segment biomechanics, neuromuscular adaptation, magnitude and direction of surgical movement, fixation strategy, and the adaptive capacity of the TMJ. The synthesised evidence indicates that condylar sag should not be viewed as a uniform technical complication but rather as a dynamic and frequently modifiable response influenced by perioperative decision-making and postoperative functional loading. Diagnostic challenges related to imaging modality and timing are highlighted, emphasising the importance of correlating radiographic findings with clinical symptoms and functional assessment to distinguish physiological adaptation from pathological instability. Preventive and management strategies are discussed across the preoperative, intraoperative, and postoperative phases, underscoring the roles of accurate condylar seating, controlled biomechanics, and structured rehabilitation. By shifting emphasis from incidence-based descriptions toward a mechanism-oriented understanding, this review provides a clinically relevant framework to minimise significant condylar sag, preserve TMJ health, and optimise long-term stability following orthognathic surgery.

## Introduction and background

Orthognathic surgery has remained a landmark in the field of dentofacial deformity that enables the re-establishment of facial harmony, functional occlusion and skeletal balance through controlled repositioning of the maxillomandibular complex [1]. Three-dimensional imaging [2], computer-planned surgery [3], and fixation are new technologies that have improved procedural accuracy and predictability of outcomes to a considerable extent. Recent comparisons of 3D virtual surgical planning versus traditional methods demonstrate improved transfer accuracy but also highlight residual sources of error relevant to condylar seating. Despite these developments, alterations achieved in the postoperative mandibular position during the postoperative period remain a clinical challenge [3]. One of these has been condylar sag, which has been associated with occlusal instability, skeletal relapse, and temporomandibular joint (TMJ) dysfunction [4]. In this review, condylar sag refers to postoperative condylar positional alteration of the proximal segment relative to the glenoid fossa, which may be transient and adaptive or may reflect clinically meaningful instability depending on mechanism and symptomatology. Clinically, it may present as early occlusal change, mandibular deviation, pain, or progressive instability. The presence of this phenomenon emphasizes that exact osteotomy and rigid fixation may not be adequate explanations of the complex biomechanical environment governing postoperative joint stability [5].

The mandibular condyle is a highly adaptive but biologically active joint system [6]. It does not depend solely on postoperative osseous alignment but is also determined by the dynamic interaction of periarticular soft tissues, neuromuscular forces, capsular tension, and disc-condylar coordination [7]. In orthognathic surgery involving mandibular osteotomies, the proximal segment is temporarily deprived of its preoperative constraints and, as such, the condyle is vulnerable to positional changes [8]. Even minor differences in muscle activity, intraoperative manipulation or fixation sequencing can affect condylar positioning within the glenoid fossa during this stage [9]. Condylar sag must not be perceived merely as a technical error but as a manifestation of disturbed biomechanical balance occurring in the proximal segment during the early healing process [10]. Systematic reviews show preexisting temporomandibular disorders alter treatment planning and risk stratification for orthognathic procedures [11].

For clarity, the proximal segment refers to the posterior mandibular segment containing the condyle after sagittal split osteotomy. Passive condylar seating describes positioning the condyle within the fossa without torsional loading or forced manipulation prior to fixation. The pterygomasseteric sling denotes the functional muscle envelope (primarily masseter and medial pterygoid) that influences proximal segment rotation and condylar loading after mandibular repositioning.

**Figure 1:**
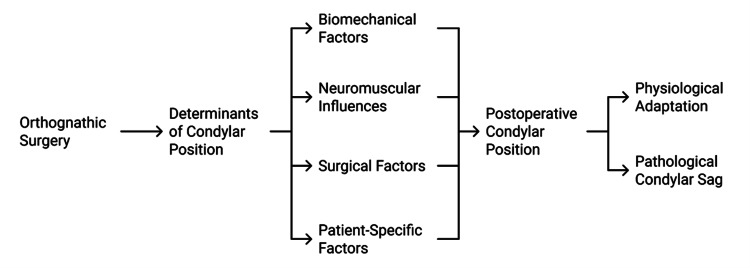
Biomechanical Determinants of Postoperative Condylar Position Created by authors

The understanding of condylar sag is not consistent in the literature, which has led to further clinical uncertainty [11]. Whereas some define it as a mechanical displacement occurring due to improper intraoperative condylar seating, others consider it a temporary adaptive reaction of the TMJ to new loading conditions [12]. This lack of consensus is further complicated by the absence of standardized diagnostic definitions, imaging protocols, and postoperative evaluation time points [13]. Immediate postoperative imaging can show apparent condylar displacement that resolves as neuromuscular adaptation occurs, whereas delayed assessment may capture remodelling rather than true instability [14]. As a result, the distinction between pathological and physiological condylar sag and normal joint adaptation remains difficult and often subjective [15]. The multifactorial nature of condylar sag is schematically modelled in Figure 1.

Rigid internal fixation was first believed to eradicate postoperative condylar instability through strict skeletal positioning [16]. However, increasing clinical evidence indicates that fixation alone is not a reliable predictor of condylar stability [17]. The TMJ is a dynamic functional unit rather than a static hinge and continuously responds to neuromuscular forces and occlusal loading [18]. Unfavourable force vectors or residual neuromuscular imbalance, even in the presence of stable fixation, can stimulate mild condylar rotation or translation, especially during the early postoperative period [19]. This perspective has shifted clinical emphasis away from fixation-centred explanations toward functional biomechanics and joint adaptation [20].

Clinical presentation of condylar sag is also complicated by patient-specific variability [21]. Some patients exhibit high adaptive capacity and can withstand extensive mandibular repositioning without long-term functional compromise, whereas others develop joint symptoms or skeletal relapse despite technically adequate surgery [22].

Despite current research, critical gaps remain in the existing literature. Evidence is not unanimous, and definitions, measurement methods, and interpretation of condylar sag show substantial variability. A large proportion of the literature remains incidence-focused or technique-comparative, with limited attention to the underlying biomechanical and neuromuscular mechanisms governing postoperative condylar behaviour. In addition, neuromuscular adaptation, surgical decision-making, and perioperative management are frequently discussed in isolation rather than as interdependent determinants of joint stability. Variability in imaging protocols and postoperative timing further obscures interpretation, particularly when distinguishing transient adaptive changes from clinically significant instability. These limitations highlight the need for an integrative, mechanism-based synthesis that explains why condylar sag occurs and how it can be anticipated, interpreted, and reduced in clinical practice.

This narrative review synthesises current evidence on the biomechanical and surgical factors influencing condylar sag following orthognathic surgery. It examines the roles of neuromuscular adaptation and perioperative surgical decision-making in postoperative condylar stability. The review also discusses diagnostic challenges and highlights clinically relevant strategies for prevention and management to support long-term TMJ health.

## Review

Methodology

Literature Identification

This narrative review synthesises English-language literature published between 2000 and 2025 that examines condylar sag in the setting of orthognathic surgery. A focused literature search was carried out across PubMed, Scopus, Web of Science, and Google Scholar using combinations of keywords such as “condylar sag,” “condylar displacement,” “orthognathic surgery,” “bilateral sagittal split osteotomy,” and “temporomandibular joint adaptation.”

Eligibility Criteria and Study Scope

Clinical studies, imaging-based investigations, and review articles that discuss postoperative condylar position, biomechanical mechanisms, surgical technique, or TMJ adaptation were considered for inclusion. Case reports, conference abstracts, animal or in vitro studies, and non-English publications were excluded.

Data Synthesis and Interpretation

The retrieved literature was synthesised qualitatively, with critical attention to methodological variability and potential sources of bias, including differences in surgical techniques, fixation methods, imaging modalities, and timing of assessment.

Limitations of the Narrative Review Design

Formal risk-of-bias scoring tools were not applied, as the intent was to identify recurring concepts and clinically meaningful insights rather than to generate quantitative comparisons, consistent with the narrative review design. Given the narrative and concept-driven nature of this review, detailed PRISMA-style search strings, screening counts, and formal risk-of-bias appraisal tools were not employed, as the objective was to synthesise mechanistic patterns rather than to conduct an exhaustive systematic evidence appraisal. Quantitative synthesis, including meta-analysis or pooled effect estimation, was not pursued due to heterogeneity in study designs, outcome definitions, imaging protocols, and follow-up timing, which precluded meaningful statistical aggregation.

Conceptual definition and types of condylar sag

Historical Technical Interpretation

Condylar sag is a postoperative finding in orthognathic surgery that has been described using numerous definitions and clinical interpretations over time [23]. Earlier literature predominantly characterised condylar sag as a technical complication resulting from insufficient intraoperative positioning of the proximal segment during mandibular osteotomy [24]. Within this framework, it was viewed as a direct mechanical error leading to inferior or anterior displacement of the condyle within the glenoid fossa following fixation [25]. However, with advances in imaging modalities and extended follow-up protocols, it became evident that postoperative condylar positional changes are not universal and cannot be explained solely by intraoperative technical factors [26].

Time-Dependent and Mechanism-Based Conceptualisation

More recent evidence supports a broader, time-dependent conceptualisation of condylar sag [7]. Positional changes may occur intraoperatively or in the immediate postoperative phase and may reflect loss of passive condylar seating, proximal segment malrotation, or fixation-related torque effects [9]. In other cases, displacement becomes apparent during the early postoperative period as neuromuscular forces and functional loading act on the repositioned mandible [12]. These early changes are often transient and may resolve as neuromuscular coordination adapts to the new skeletal configuration [14].

Late Adaptive and Degenerative Changes

In contrast, long-term alterations in condylar position are more likely to represent adaptive remodelling or, in some cases, degenerative joint responses rather than true mechanical sag [16]. Failure to differentiate between early transient changes and late adaptive or degenerative patterns has contributed to inconsistent reporting and overinterpretation of postoperative imaging findings [18].

Clinical Relevance and Diagnostic Implications

**Figure 2:**
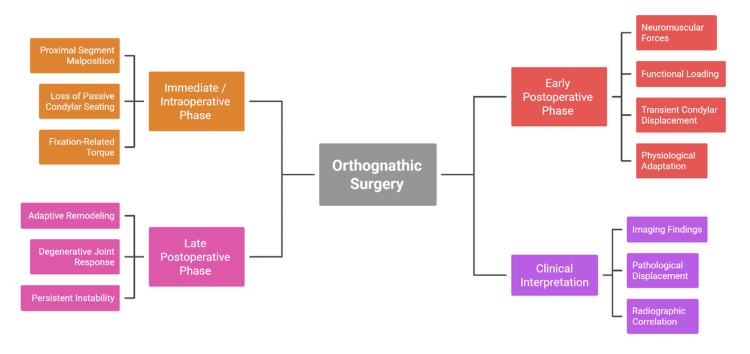
Temporal Framework for Postoperative Condylar Changes Created by authors

Accurate distinction between physiological adaptation and clinically significant instability is therefore essential [11]. Not all postoperative condylar positional changes are pathological, and premature reliance on early imaging may lead to unnecessary concern or intervention [15]. A mechanism-based understanding that integrates timing, biomechanics, and functional adaptation provides a more reliable framework for clinical interpretation [10]. From this perspective, condylar sag is not viewed as a singular complication but as a multifactorial response within a dynamic joint system [6]. Figure 2 schematically illustrates patterns of postoperative condylar behaviour over time.

Proximal segment biomechanics after osteotomy

Immediate Intraoperative Instability

As a primary determinant of postoperative condylar position, the biomechanical behaviour of the proximal segment following mandibular osteotomy plays a central role [27]. In bilateral sagittal split osteotomy, the proximal segment is momentarily relieved of its preoperative constraints, resulting in a period of intrinsic instability [28]. Proximal segment reseating within the glenoid fossa is therefore essential once the distal segment has been repositioned [14]. Even minimal alignment errors at this stage can translate into measurable condylar displacement [29,30].

Osteotomy Design and Segment Mobility

Several biomechanical factors influence proximal segment behaviour [7]. The design and execution of the osteotomy determine the degree of segment mobility, with broader osteotomy cuts and greater disruption of periosteal and muscular attachments increasing freedom of movement [12]. While increased mobility may facilitate mandibular repositioning, it also raises the risk of unintended rotation or translation if adequate control is not maintained [9]. Such rotations may occur across multiple planes depending on the magnitude and direction of mandibular movement, potentially compromising passive condylar seating [18].

Fixation Application and Torque Effects

Intraoperative torque represents another important contributor to condylar instability [15]. Torque may be introduced manually through inadequate plate adaptation or uneven screw tightening [16]. Although these forces may not be evident intraoperatively, they can manifest postoperatively once neuromuscular activity resumes [11]. Rigid fixation, while necessary for skeletal stability, may inadvertently stabilise the proximal segment in an unfavourable position if applied before accurate condylar seating is ensured [17]. Accordingly, fixation should be regarded as a means of maintaining condylar position rather than determining it [21]. Continuous attention to proximal segment biomechanics during surgery, therefore, remains fundamental to preventing mechanism-driven condylar sag [25].

Muscular and neuromuscular influences on condylar position

Early Postoperative Neuromuscular Effects

Neuromuscular and muscular factors exert substantial influence on postoperative condylar behaviour and frequently account for positional variations independent of surgical technique [29]. The pterygomasseteric sling plays a dominant role in mandibular posture and condylar loading [4]. Surgical repositioning alters muscle length-tension relationships, generating new force vectors acting on the proximal segment [17]. During the early postoperative period, these altered forces may be unevenly distributed, contributing to transient condylar displacement [8].

Muscle Memory and Adaptive Stabilisation

Muscle memory further influences condylar positioning after surgery [23]. Muscles conditioned to maintain the preoperative mandibular posture may initially generate forces favouring a return toward the original skeletal relationship [2]. This phenomenon helps explain why condylar position may fluctuate during the weeks following surgery despite technically optimal intraoperative positioning [30]. With time, neuromuscular adaptation allows the musculature to accommodate the new mandibular position, often resulting in spontaneous stabilisation of the condyle [11].

Functional Loading and Clinical Implications

This interaction between neuromuscular adaptation and mechanical fixation highlights the limitations of a strictly structural definition of condylar stability [6]. Fixation does not eliminate functional loading arising from mastication, speech, or parafunctional activity during bone healing [6]. Early functional loading in the presence of altered occlusion or residual muscular imbalance may therefore produce positional changes that reflect adaptive processes rather than pathological instability [27]. Consequently, postoperative functional management, including occlusal guidance and physiotherapy, constitutes an integral component of condylar stability rather than an auxiliary intervention [14]. Table 1 summarises key neuromuscular and functional influences on postoperative condylar position.

**Table 1:** Neuromuscular and Functional Influences on Postoperative Condylar Behaviour.

Category	Mechanism	Timing	Clinical Effect on Condyle	Management Implication	Reference
Neuromuscular factors	Altered muscle force patterns independent of surgical technique	Early postoperative period	Positional changes not explained by fixation or osteotomy	Requires functional rather than purely structural interpretation	[29]
Muscular support system	The pterygomasseteric sling governs mandibular posture and condylar loading.	Immediate to early postoperative phase	Influences the direction and magnitude of condylar loading	Attention to muscle balance during planning and recovery	[4]
Muscle biomechanics	Changes in muscle length–tension relationships after repositioning	Immediate postoperative phase	New force vectors act on the proximal segment	Anticipation of transient displacement	[17]
Force distribution	Uneven neuromuscular loading	Early postoperative phase	Transient condylar displacement	Conservative monitoring favoured	[8]
Muscle memory	Retained neuromuscular patterns from the preoperative state	Weeks following surgery	Tendency toward original mandibular position	Emphasises the role of adaptation over time	[23]
Relapse tendency	The elevator muscle pulls toward the preoperative skeletal relationship	Early healing phase	Temporary alteration in condylar position	Gradual functional re-education	[2]
Neuromuscular adaptation	Progressive accommodation to the new mandibular position	Intermediate postoperative period	Spontaneous stabilisation of the condyle	Supports delayed definitive assessment	[10]
Structural–functional interaction	Fixation does not eliminate functional loading	Throughout healing	Condylar changes despite stable fixation	Highlights the limits of fixation-centered view	[19]
Functional loading	Mastication, speech, parafunction	Early to late postoperative period	Adaptive rather than pathological displacement	Functional management essential	[6]
Postoperative management	Occlusal guidance and physiotherapy	Recovery phase	Enhances condylar stability	Integral component of treatment	[14]

TMJ adaptive capacity

Physiological Adaptive Potential

The TMJ is a highly specialised joint with substantial functional adaptive capacity [31]. It responds dynamically to changes in occlusal relationships, mandibular positioning, and muscular forces, unlike purely load-bearing joints [32]. This adaptive potential is central to postoperative condylar behaviour after orthognathic surgery and explains why condylar positional changes do not necessarily translate into clinical pathology [33]. Within physiological limits of mechanical stress, the joint may remodel in a manner that preserves function and long-term stability [34]. The disc-condyle-fossa complex is a critical component of this adaptive response, distributing joint loads during mandibular function [35]. Orthognathic surgery alters mandibular position and thereby modifies the direction and magnitude of forces transmitted through this complex [18]. When disc position and condylar seating are maintained, adaptive remodelling may occur without adverse sequelae [12]. However, this balance may be disrupted by excessive loading, altered neuromuscular coordination, or underlying joint pathology, reducing the effectiveness of adaptive compensation [27]. Under such circumstances, condylar positional changes may exceed physiological adaptation and progress toward maladaptive remodelling or degenerative change [21].

Limits of Adaptation and Maladaptive Responses

Despite its adaptive capacity, the TMJ has finite biological tolerance. Lateral or prolonged loading may exceed this tolerance, producing capsular strain, disc displacement, or surface remodelling of the condyle [25]. Patients with a history of temporomandibular disorders and those with reduced joint volume or disc pathology appear particularly prone to maladaptive responses [19]. These findings indicate that postoperative condylar behaviour reflects a balance between biomechanical loading and individual adaptive capacity rather than being solely determined by surgical precision [10]. This understanding supports caution when interpreting postoperative imaging findings [23]. Apparent condylar displacement, particularly within the first postoperative months, may represent a transient adaptive phenomenon rather than surgical failure [16]. In contrast, chronic or progressive positional change accompanied by functional symptoms warrants closer evaluation [28]. Recognition of the limits of TMJ adaptability reinforces the need for individualized surgical planning and clinically guided postoperative interpretation rather than reliance on radiographic alignment alone [11].

Surgical movement magnitude and direction

Biomechanical Impact of Movement Magnitude

The magnitude and direction of mandibular movements during orthognathic surgery impose substantial biomechanical demands on postoperative condylar stability [36]. Larger skeletal movements increase biomechanical loading on the proximal segment, periarticular musculature, and joint capsule, thereby challenging the adaptive capacity of the TMJ [37]. As the extent of movement increases, the likelihood that adaptive mechanisms will be exceeded correspondingly rises.

Directional Force Vectors and Condylar Loading

Different mandibular movements generate distinct force vectors that alter condylar loading patterns, resulting in variable effects on joint stability [38]. Mandibular advancement and rotational movements are commonly associated with increased tension within the pterygomasseteric sling and capsular structures, producing anteriorly directed forces that may interfere with condylar seating during the early postoperative phase [39]. In contrast, mandibular setback procedures tend to generate compressive forces within the glenoid fossa, which may be stabilizing when moderate but can become detrimental if excessive joint loading occurs [40]. These directional differences demonstrate that movement magnitude alone is insufficient to predict postoperative condylar behaviour; the direction of force application and stress distribution are equally critical determinants [22].

Threshold Effects and Surgical Risk Factors

Clinical evidence suggests that beyond certain thresholds of skeletal movement, the risk of condylar instability increases as neuromuscular adaptation and joint remodelling capacity are exceeded [17]. This risk is further amplified in cases involving large movements, prolonged operative time, extensive periosteal stripping, or complex bimaxillary procedures [29]. Accordingly, heightened intraoperative and postoperative vigilance is warranted, particularly in patients with pre-existing TMJ vulnerability or limited adaptive reserve [18].

Strategic Planning to Reduce Biomechanical Load

Strategic surgical planning offers opportunities to mitigate these risks [24]. Redistribution of skeletal correction between the maxilla and mandible, staged surgical approaches, or modest reduction in movement magnitude can decrease biomechanical stress on the TMJ without compromising overall treatment objectives [16]. Preoperative evaluation of joint morphology and function further informs individualized decision-making, allowing surgical movements to be tailored to the patient’s adaptive capacity [27]. From this perspective, optimal condylar stability is achieved not through maximal skeletal correction in a single manoeuvre, but through balancing functional demands with biological tolerance [21]. Table 2 summarises the effects of movement magnitude and direction on postoperative condylar stability.

**Table 2:** Influence of Mandibular Movement Magnitude and Direction on Condylar Stability.

Surgical Variable	Biomechanical Effect	Force Direction/Pattern	Risk to Condylar Stability	Clinical Consideration	Reference
Magnitude of mandibular movement	Increased biomechanical demand on the proximal segment and joint structures	Multidirectional loading	Greater likelihood of challenged adaptive mechanisms	Limit excessive single-stage movements when possible	[16]
Large skeletal movements	Elevated stress on the musculature and joint capsule	Increased tensile and compressive forces	Higher risk of postoperative instability	Careful intraoperative control is required	[7]
Type of mandibular movement	Altered condylar loading patterns	Advancement vs setback–specific vectors	Differential effects on joint stability	Movement direction must be considered independently	[38]
Mandibular advancement	Increased tension in the pterygomasseteric sling and capsule	Predominantly anteriorly directed forces	Potential alteration of condylar seating	Monitor early postoperative condylar behaviour	[39]
Mandibular setback	Increased compressive loading within the glenoid fossa	Posterior and superior compressive forces	Risk of joint overload if excessive	Controlled setback magnitude is critical	[40]
Force direction relevance	Stress distribution outweighs magnitude alone	Direction-dependent joint loading	Unpredictable condylar response if ignored	Directional biomechanics must guide planning	[22]
Movement thresholds	Adaptive capacity may be exceeded	Sustained neuromuscular loading	Increased risk of condylar instability	Identify patient-specific tolerance limits	[17]
Combined surgical burden	Prolonged surgery and extensive dissection	Cumulative biomechanical stress	Amplified instability risk	Minimise operative time and tissue trauma	[29]
Patient vulnerability	Preexisting TMJ pathology or limited reserve	Reduced adaptive response	Higher likelihood of adverse outcomes	Requires an individualised surgical strategy	[18]
Surgical planning strategy	Redistribution or staging of correction	Balanced force application	Reduced joint stress	Supports long-term stability	[24]
Movement moderation	Reduced magnitude per surgical step	Lower peak biomechanical load	Enhanced adaptive response	Consider staged or combined approaches	[16]
Preoperative joint assessment	Evaluation of joint morphology and function	Baseline adaptive capacity	Improved risk stratification	Tailor surgical movement to the patient	[27]
Surgical philosophy	Balance correction with biological tolerance	Controlled force environment	Optimised condylar stability	Avoid maximal correction in a single manoeuvre	[21]

Fixation techniques and condylar stability

Rigid Versus Semi-Rigid Fixation

Skeletal alignment is maintained through fixation techniques after orthognathic surgery; however, their effect on postoperative condylar stability is frequently overestimated [41]. Rigid and semi-rigid fixation systems provide different degrees of stability and flexibility, yet no single fixation method inherently guarantees accurate condylar positioning [42]. Fixation primarily preserves the spatial relationship present at the time of its application rather than establishing condylar stability independently [43]. Rigid fixation offers greater resistance to micromovement and supports predictable bone healing [44]. Nevertheless, rigid fixation may stabilise the proximal segment in an unfavourable position if condylar seating is not achieved before fixation [45]. Semi-rigid fixation allows limited adaptive movement, which may support early neuromuscular adjustment, but it may also permit unwanted displacement if biomechanical forces are not adequately controlled [18]. Accordingly, the clinical effectiveness of fixation depends less on stiffness and more on accurate proximal segment positioning prior to stabilisation [27].

Hardware Design, Sequencing, and Torque Effects

Hardware design and application technique further influence postoperative condylar behaviour [9]. Plate configuration, screw placement, and fixation sequencing may introduce unintended rotational or translational forces on the proximal segment [30]. Inadequate plate adaptation or uneven screw tightening may cause subtle positional changes that become apparent only after postoperative function resumes [16]. These considerations underscore the importance of a deliberate fixation strategy rather than routine hardware application [21].

Fixation as a Stabilising Tool Rather Than a Corrective Measure

Fixation should be regarded as a stabilising adjunct rather than a corrective mechanism for proximal segment malposition [6]. No fixation system can compensate for inaccurate proximal segment alignment or inadequate condylar seating [33]. Therefore, optimal outcomes depend on achieving accurate intraoperative condylar positioning, followed by fixation to maintain this relationship throughout healing [14]. When interpreted within a broader biomechanical and functional framework, long-term condylar stability reflects surgical precision, controlled biomechanics, and appropriate postoperative functional adaptation rather than hardware selection alone [25].

Intraoperative condylar positioning and technical pitfalls

Technical Control and Segment Positioning

One of the most influential and modifiable determinants of postoperative condylar stability is intraoperative management of the proximal segment [46]. Even with meticulous preoperative planning, technical shortcomings at this stage may predispose the condyle to positional instability that later manifests as condylar sag [47]. Inadequate proximal segment control, unrecognised segmental malrotation, and excessive reliance on occlusal positioning as a surrogate for condylar seating represent common intraoperative pitfalls [48]. These factors may act independently or synergistically to compromise accurate condylar positioning [49].

Proximal Segment Malrotation and Intraoperative Risk Factors

Proximal segment malrotation is a significant yet frequently under-recognised contributor to postoperative instability [10]. Following mandibular osteotomy, release of periosteal and muscular constraints markedly increases segment mobility, rendering the proximal segment susceptible to rotation across multiple planes [27]. If such malrotation is not identified and corrected prior to fixation, the condyle may become stabilised in a non-physiological position [18]. This risk is heightened in cases involving large skeletal movements, bimaxillary procedures, or prolonged operative times, where repeated manipulation may gradually alter segment alignment [36]. When assessment relies primarily on occlusal relationships, these rotational discrepancies may remain undetected, underscoring the limitations of occlusion-based verification [12].

Limitations of Occlusion-Based Verification

Use of occlusion as the principal determinant of mandibular positioning remains a widespread technical limitation [41]. Although occlusal assessment provides essential information regarding distal segment alignment, it does not consistently reflect condylar position within the glenoid fossa [7]. Under general anaesthesia, muscle relaxation may temporarily obscure discrepancies between occlusal stability and joint alignment [22]. Consequently, a clinically acceptable occlusion may coexist with subtle yet significant condylar displacement [30]. Failure to independently evaluate proximal segment seating permits malposition to persist until postoperative functional loading reveals instability [15].

Verification Strategies and Adjunctive Aids

**Figure 3:**
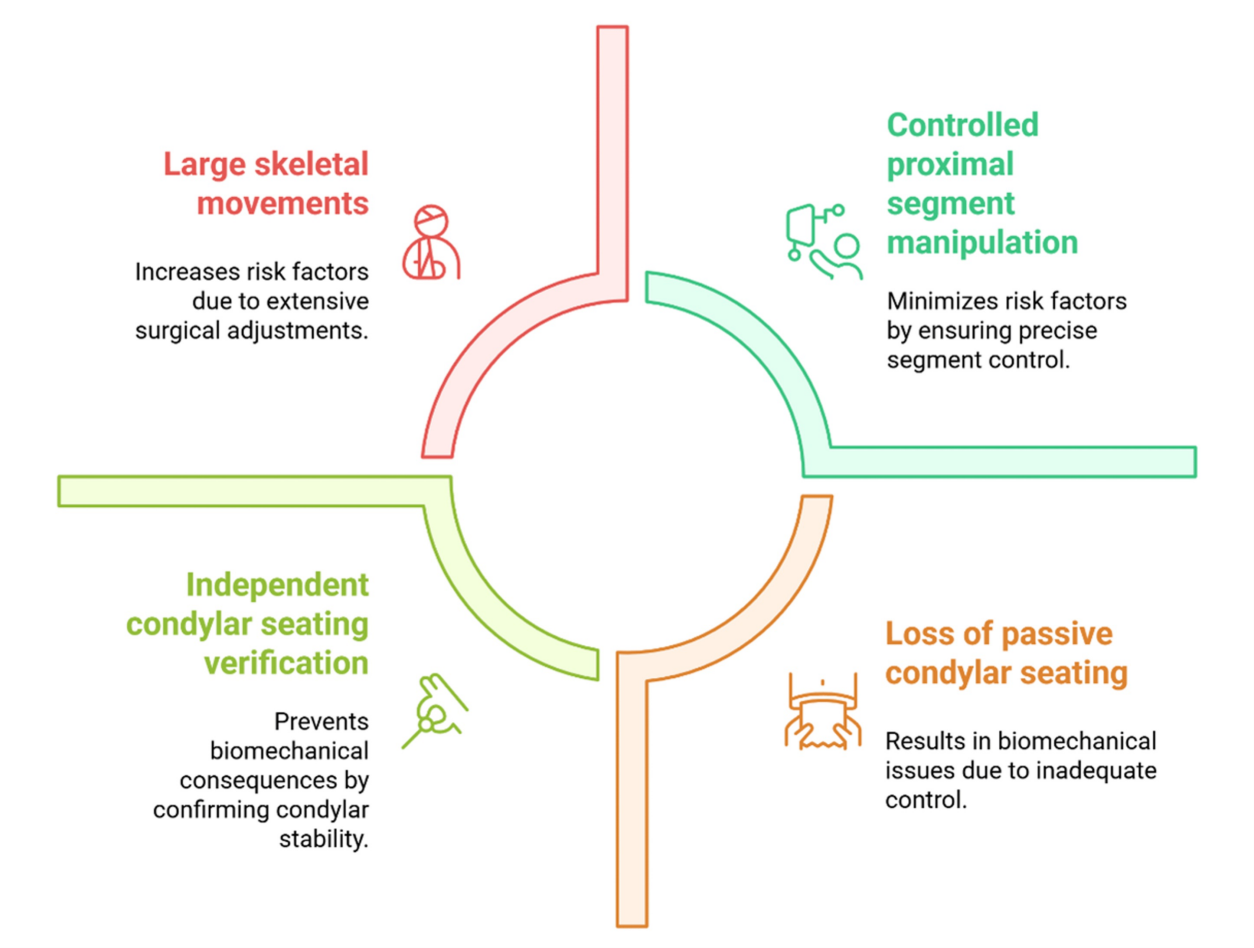
Intraoperative Factors Influencing Proximal Segment Control Created by authors

Accurate confirmation of condylar seating before definitive fixation is therefore essential [9]. This process requires deliberate assessment of proximal segment position through controlled mandibular manipulation and tactile feedback [24]. Condylar positioning devices and intraoperative splints may enhance reproducibility and reduce subjective variability, particularly in complex or high-risk cases [33]. However, their effectiveness depends on appropriate application and surgical experience [19]. Such adjunctive tools do not replace biomechanical understanding or sound intraoperative judgement [6]. Figure 3 illustrates modifiable intraoperative factors influencing postoperative condylar stability.

Imaging modalities and diagnostic timing

Imaging has significantly improved assessment of postoperative condylar position; however, interpretation remains highly dependent on the selected modality and the timing of acquisition [42]. The most commonly used complementary modalities are cone-beam computed tomography (CBCT) and magnetic resonance imaging (MRI) [10]. CBCT provides high-resolution visualization of osseous structures, enabling assessment of condylar position, rotation, and its relationship to joint spaces [31]. MRI, in turn, enables evaluation of soft-tissue structures, including disc position, effusion, and inflammatory changes, thereby providing information relevant to functional joint adaptation [18]. Accordingly, imaging findings should be interpreted in conjunction with the clinical presentation rather than in isolation [49]. A key challenge is distinguishing transient adaptive changes from clinically meaningful instability, for which timing is critical [12]. Early postoperative imaging can reflect muscular relaxation, postoperative oedema, or altered functional loading, which may mimic condylar displacement [36]. In most patients, subsequent follow-up demonstrates neuromuscular re-coordination and restoration of functional balance [9]. Therefore, early imaging alone may contribute to overdiagnosis of condylar sag and potentially unnecessary intervention [27].

Delayed imaging, conversely, may capture remodelling rather than true postoperative displacement [44]. Changes in the condylar surface measured months after surgery are often adaptive responses to sustained loading rather than evidence of intraoperative positional error [21]. Interpretation is further complicated by variability in imaging protocols, head positioning, and reference landmarks, which limit reliable longitudinal comparison and contributes to inconsistent reporting across studies [15]. Accurate diagnosis of condylar sag requires integrating imaging with clinical assessment [33]. Persistent positional change accompanied by pain, mandibular deviation, or occlusal instability warrants closer evaluation, whereas asymptomatic positional variation, particularly in the immediate postoperative period, can often be managed conservatively [5]. Routine postoperative scanning is not recommended; a selective, indication-based imaging strategy improves diagnostic specificity and reduces misclassification of physiological adaptation as pathology [40].

Prevention and clinical management framework

Preoperative Risk Stratification

Condylar sag requires a multiphase approach that integrates biomechanical, surgical, and functional considerations across the perioperative period [34]. Prevention begins preoperatively with careful assessment of TMJ status, joint morphology, and individual adaptive capacity [7]. Identification of pre-existing joint pathology or limited biological reserve may justify modifications in surgical planning, including reduction in movement magnitude or redistribution of correction between skeletal segments [46].

Intraoperative Control and Fixation Strategy

Intraoperatively, prevention relies on meticulous control of the proximal segment and accurate condylar seating before fixation [12]. The condylar position should be independently verified, fixation applied in a controlled manner, and excessive torque avoided [29]. Positioning devices or splints may assist in selected high-risk cases when used with an appropriate biomechanical rationale [41].

Postoperative Monitoring and Rehabilitation

Postoperatively, imaging should be employed selectively based on symptoms or risk profile, with findings interpreted in conjunction with clinical assessment. Conceptualizing condylar sag as a spectrum of biomechanical and neuromuscular adaptation rather than a single technical complication supports individualized prevention and management strategies [16]. Through anticipatory planning, intraoperative precision, and targeted postoperative rehabilitation, the risk of clinically significant condylar sag can be reduced while preserving long-term TMJ health [44]. Table 3 summarises unified perioperative measures supporting condylar stability.

**Table 3:** Phase-Specific Strategies for Prevention and Management of Condylar Sag.

Perioperative Phase	Key Focus	Mechanism/Action	Intended Effect on Condyle	Clinical Implementation	Reference
Overall strategy	Comprehensive approach	Integration of biomechanical, surgical, and functional factors	Reduction of condylar instability risk	Phase-specific planning across the perioperative timeline	[34]
Preoperative phase	TMJ assessment	Evaluation of joint status, morphology, and adaptive capacity	Identification of vulnerability to instability	Risk stratification and individualised planning	[7]
Surgical planning	Modification of correction	Adjustment of movement magnitude or redistribution across segments	Reduced biomechanical stress on TMJ	Avoid excessive single-stage correction	[46]
Intraoperative phase	Proximal segment control	Accurate condylar seating before fixation	Maintenance of physiological condylar position	Active verification beyond occlusal cues	[12]
Fixation technique	Fixation sequencing	Controlled application and avoidance of excessive torque	Prevention of fixation-induced malposition	Deliberate fixation strategy	[29]
Adjunctive support	Positioning aids	Use of splints or positioning devices in high-risk cases	Enhanced stability during fixation	Applied with biomechanical understanding	[41]
Surgical principle	Role of fixation	Precision and judgment over hardware reliance	Improved condylar seating accuracy	Emphasis on surgical technique	[18]
Postoperative phase	Functional adaptation	Promotion of neuromuscular equilibrium	Stabilisation of condylar position	Early functional management	[12]
Occlusal management	Occlusal splints	Temporary guidance during early healing	Reduced asymmetric loading	Short-term supportive use	[9]
Rehabilitation	Physiotherapy	Restoration of muscle coordination and joint mobility	Support adaptive remodelling	Structured rehabilitation protocols	[22]
Mobilization	Functional loading	Early, controlled mandibular movement	Reduced risk of persistent instability	Gradual return to function	[31]
Care model	Multidisciplinary approach	Collaboration among surgical and allied specialities	Coordinated functional recovery	Integrated treatment planning	[5]
Team coordination	Interdisciplinary care	Surgeon-orthodontist-physiotherapist collaboration	Optimised occlusal and skeletal outcomes	Continuous communication	[39]
Conceptual framework	Reframing condylar sag	Viewing it as a modifiable biomechanical response	Proactive prevention and tailored care	Shift from a complication-based mindset	[16]
Long-term goal	Outcome preservation	Anticipatory planning and targeted rehabilitation	Sustained TMJ health and stability	Long-term follow-up strategy	[44]

Limitations and future directions

The existing knowledge about condylar sag after orthognathic surgery is limited by several methodological and conceptual flaws. The lack of a standardised definition contributes to inconsistent diagnostic thresholds and inconsistent interpretation of studies. Imaging modalities, referral landmarks, and the time of postoperative evaluation also contribute to heterogeneity, increasing the limitation of comparing and synthesising findings. Moreover, confounding variables that are not isolable are variations in surgical technique, fixation techniques and experience of the operators. Most existing studies are based on a limited period of follow-up, which limits the understanding of TMJ adaptation in the long term, and all studies are mainly observational, which makes it difficult to draw causal conclusions. The difference between physiological adaptation and clinically significant instability, thus, is a difficult issue to draw and is one that can lead to excessive and inadequate estimation of the actual condylar sag.

The next step in improving consistency and reproducibility in the area should be the development of standardised diagnostic criteria and imaging protocols in future research. Properly designed longitudinal studies that have a long follow-up would be required to shed light on the natural process of condylar postoperative changes and their functional implications. The integration of biomechanical models, three-dimensional models, and AI-based surgical planning could improve the condylar behaviour prediction and allow risk stratification of patients. It will be necessary to place more focus on the functional outcomes and patient-reported measures in comparison to the positional measures to further elucidate clinical relevance and maximise the long-term TMJ health.

## Conclusions

The current narrative review indicates that the phenomenon of condylar sag after an orthognathic surgery is rather a complex and multifactorial biomechanical reaction than an irretrievable postoperative complication. The produced evidence suggests that postoperative condylar behaviour is regulated by the interactions between proximal segment biomechanics, neuromuscular adjustments, the magnitude and direction of surgical motion, fixation strategy, and the adaptive ability of TMJ. Proper intraoperative condylar seating, controlled fixation sequencing and lack of excessive biomechanical stress are identified as the most important factors to determine early stability after surgery and long-term joint adaptation is maintained by structured functional rehabilitation. Notably, the postoperative condylar displacement is not always an indication of pathological instability; temporary positional alterations can belong to the physiological adaptation and need to be considered in connection with the timeline, clinical manifestations, and functional results. The overdependence on radiographic examination alone can hence result in misclassification and unnecessary treatment. Mechanism-based, patient-focused, and interdisciplinary model of patient care incorporating a combination of careful surgical practice with postoperative management strategies is best placed to reduce clinically significant condylar sag. Clinicians can increase the health of TMJ and enhance its stability in the long term after orthognathic surgery by switching the focus of incidence-based interpretation to functional and biomechanical insights.
